# Beyond Pain Relief: An In-Depth Review of Vertebral Height Restoration After Balloon Kyphoplasty in Vertebral Compression Fractures

**DOI:** 10.7759/cureus.46124

**Published:** 2023-09-28

**Authors:** Siddharth K Patel, Sohael Khan, Ventaktesh Dasari, Suvarn Gupta

**Affiliations:** 1 Orthopaedics, Jawaharlal Nehru Medical College, Datta Meghe Institute of Higher Education and Research, Wardha, IND

**Keywords:** quality of life, functional recovery, clinical outcomes, vertebral height restoration, vertebral compression fractures, balloon kyphoplasty

## Abstract

This comprehensive review delves into the intricate landscape of vertebral height restoration after balloon kyphoplasty in cases of vertebral compression fractures. With a comprehensive examination of procedural intricacies, radiological evaluations, clinical outcomes, and influential factors, a nuanced comprehension unfolds. Beyond its immediate alleviation of pain, vertebral height restoration emerges as a linchpin in enhancing spinal alignment, fostering functional recuperation, and augmenting the overall quality of life. This review underscores the pivotal role of balloon kyphoplasty, transcending its mere medical utility to become a conduit for renewed independence and well-being among individuals grappling with vertebral compression fractures. The ongoing advancements in medical science and the continued pursuit of research stand poised to amplify the significance of vertebral height restoration, manifesting a promising horizon for individuals seeking respite from pain, a revitalised capacity for movement, and a life unburdened by its constraints.

## Introduction and background

Vertebral compression fractures (VCFs) represent a prevalent and debilitating condition, primarily affecting the ageing population and individuals with compromised bone health. These fractures often result from osteoporosis, trauma, or metastatic disease, leading to vertebral body collapse, pain, and a diminished quality of life. Vertebral compression fractures can significantly burden patients' physical well-being, mobility, and independence [1,2].

Balloon kyphoplasty, a minimally invasive vertebral augmentation technique, has become an effective intervention for managing VCFs. This procedure involves the percutaneous insertion of specialised balloons into the fractured vertebra, followed by inflation to create a void for cement injection. Subsequent cement augmentation restores vertebral height, stabilises the fracture, and alleviates pain. The procedure's minimally invasive nature allows for quicker recovery and reduced hospitalisation time compared to traditional surgical interventions [3,4].

While pain relief is crucial to VCF management, the significance of vertebral height restoration extends beyond immediate pain mitigation. Maintaining vertebral height is essential for preserving spinal alignment, preventing kyphotic deformities, and ensuring optimal load distribution across the spinal column. Vertebral height restoration can be pivotal in improving patients' functional capacity, minimising disability, and enhancing the overall quality of life [2,5].

The primary objective of this comprehensive review is to delve into the multifaceted dimensions of vertebral height restoration after balloon kyphoplasty in VCFs. Beyond merely addressing pain relief, this review aims to critically assess the clinical, radiological, and functional outcomes associated with vertebral height restoration. By examining various research studies, clinical trials, and real-world experiences, this review provides an in-depth understanding of the benefits, challenges, and implications of vertebral height restoration through balloon kyphoplasty. Through the synthesis of existing literature, this review aims to guide clinicians, researchers, and healthcare practitioners in making informed decisions regarding managing VCFs and optimising patient outcomes.

## Review

Balloon kyphoplasty: mechanism and technique

Explanation of Balloon Kyphoplasty Procedure

Balloon kyphoplasty is a specialized procedure designed to restore vertebral height and alleviate pain caused by VCFs. It involves a series of carefully orchestrated steps that combine surgical precision with image-guided techniques. The procedure begins with the patient in a prone or lateral position under local or general anaesthesia. Small incisions are made through which specialised instruments are introduced into the affected vertebral body [6].

Step-by-Step Description of the Technique

Access and balloon insertion: A trocar is inserted into the fractured vertebral body through a small incision under fluoroscopic guidance. The trocar is a pathway for introducing the inflatable balloon [7].

Balloon placement: Once the trocar is in place, an inflatable balloon is threaded and positioned within the fractured vertebra [8].

Balloon inflation: The balloon is gradually inflated to create a void within the vertebral body, restoring its original height and shape. This step not only lifts the collapsed bone but also helps to create space for the subsequent cement injection [9].

Cement injection: After achieving the desired vertebral height, the balloon is deflated and removed, leaving a cavity within the vertebral body. Medical-grade bone cement is injected into the cavity, filling the void and stabilising the fractured vertebra [10].

Cement solidification: The injected cement solidifies quickly, providing structural support and stability to the treated vertebral body. This process helps prevent further collapse and minimises the risk of subsequent fractures [11].

Incision closure: Following the completion of the cement injection, the instruments are removed, and the incisions are closed. The procedure typically takes one to two hours per treated vertebra [4].

Role of Balloon Inflation in Vertebral Height Restoration

The inflation of the balloon during the kyphoplasty procedure is a pivotal step in achieving vertebral height restoration. The balloon creates a void or cavity within the bone by gently expanding the collapsed vertebral body. This cavity not only facilitates the elevation of the collapsed bone fragments but also provides a space into which the cement can be injected. The balloon’s controlled expansion helps reduce the kyphotic deformity and restore the vertebra's natural height and alignment. The balloon inflation process is integral to restoring optimal vertebral height and maintaining spinal stability and overall function [12]. The mechanism and technique of Balloon kyphoplasty form the foundation for successful vertebral height restoration, ensuring improved spinal alignment, pain relief, and enhanced patient outcomes.

Clinical assessment of vertebral height restoration

Patient Selection Criteria for Balloon Kyphoplasty

Painful vertebral compression fractures: The primary indication for balloon kyphoplasty is the presence of painful vertebral compression fractures. These fractures occur when the vertebrae, the small bones of the spine, collapse or become compressed. This can result in intense pain due to nerve irritation and instability of the spine. Candidates should have fractures causing significant pain and affecting their quality of life [4].

Inadequate response to conservative treatments: Before considering balloon kyphoplasty, patients should have tried conservative treatments such as pain medications, rest, and physical therapy. Balloon kyphoplasty is an alternative if these treatments have not provided sufficient relief from pain or improved functionality [13].

Minimal spinal deformity: Balloon kyphoplasty is most effective for patients with minimal spinal deformity. It aims to restore the height of the collapsed vertebra and stabilise the spine. Other surgical options might be more appropriate if the deformity is too severe [3].

Adequate bone quality for cement augmentation: During the balloon kyphoplasty procedure, a balloon is inserted into the fractured vertebra and inflated to create a cavity. This cavity is then filled with bone cement to stabilise the bone. Candidates should have sufficient bone quality to support the injection of the cement and ensure that it forms a stable bond [14].

Absence of contraindications: Contraindications make a patient unsuitable for a particular medical procedure. In the case of balloon kyphoplasty, contraindications might include active infections in the spine, bleeding disorders that could complicate the procedure, or other medical conditions that would increase the risks associated with the surgery [15].

Preoperative Evaluation and Imaging

Preoperative evaluation and imaging considerations: Before balloon kyphoplasty, patients undergo a comprehensive preoperative evaluation and imaging assessment to ensure the procedure's suitability and safety. This evaluation process involves several steps that help medical professionals gather crucial information about the patient's medical history, current condition, and the specific characteristics of the spinal fracture they are dealing with. The primary goals of this evaluation are to identify potential risk factors, determine the severity of the vertebral fracture, assess overall spinal health, and evaluate the patient's bone quality [16].

Medical history review: The medical history review involves a detailed examination of the patient's past medical records and personal medical history. This helps identify any underlying medical conditions that might affect the patient's eligibility for the procedure or influence the treatment plan. Factors such as osteoporosis, previous spinal surgeries, chronic medical conditions, and medication use are considered. This step aids in understanding the patient's overall health and provides insights into potential risks associated with the procedure [17].

Physical examination: A thorough physical examination assesses various aspects of the patient's spinal health. The neurologic function is evaluated to check for any signs of nerve compression or damage resulting from the vertebral fracture. Spinal alignment is assessed to determine if the fracture causes any deformity. The severity of pain and its impact on the patient's daily life are also evaluated. This examination helps the medical team understand the immediate physical effects of the fracture and guides the treatment approach [18].

Imaging studies: Imaging plays a crucial role in assessing the severity of the vertebral fracture and the overall condition of the spine. Different imaging modalities are used, including X-rays, CT scans, and MRI. X-rays provide a basic view of the fracture and help evaluate vertebral collapse and alignment. The CT scans offer detailed cross-sectional images of the fracture, aiding in precise measurement and planning of the procedure. An MRI can reveal additional information about soft tissue structures and nerve involvement [19].

Bone density assessment: Bone density assessment, often performed through a dual-energy X-ray absorptiometry (DEXA) scan, is used to measure bone mineral density. This assessment helps determine the quality and strength of the patient's bones, particularly in osteoporosis or other conditions that affect bone density. It provides valuable information about the patient's susceptibility to future fractures and aids in tailoring the treatment plan to address underlying bone health issues [20].

Clinical Outcomes Assessment Post-Balloon Kyphoplasty

Pain reduction: One of the primary goals of balloon kyphoplasty is to alleviate pain caused by vertebral compression fractures. To quantify the extent of pain reduction, healthcare providers use validated pain assessment tools such as the Visual Analogue Scale (VAS) or the Numeric Rating Scale (NRS). These scales allow patients to rate their pain intensity from 0 to 10, with 0 indicating no pain and 10 indicating the worst pain imaginable. Patients are typically asked to rate their pain before the procedure and then at follow-up appointments after the balloon kyphoplasty. The difference between the pre-procedure and post-procedure scores provides a measurable indication of pain reduction [21].

Reduction in pain medication consumption: Another critical aspect of assessing the effectiveness of balloon kyphoplasty is evaluating whether there is a decrease in the consumption of pain medications. Patients with vertebral compression fractures often require pain-relieving medications to manage their discomfort. After the procedure, if the patient's reliance on analgesic medication decreases, it indicates a successful pain reduction resulting from the kyphoplasty. Tracking the dosage and frequency of pain medications before and after the procedure helps quantify this reduction [1].

Quality of life improvement: Beyond pain reduction, balloon kyphoplasty aims to improve the patient's overall quality of life, which can be negatively affected by chronic pain and functional limitations. To assess this improvement, standardised questionnaires are used. Two standard questionnaires are the Short Form 36 (SF-36) and the EuroQol 5-Dimension (EQ-5D). These questionnaires cover various dimensions of a patient's quality of life, including physical functioning, emotional well-being, social interactions, and more. Patients complete these questionnaires before the procedure and then at follow-up visits, providing a quantifiable measure of improvements in their daily lives [22].

Mobility and Functional Recovery Beyond Pain Relief, Assessing Improvements in Mobility and Functionality Is Crucial

Measurement of functional mobility using validated scales: Functional mobility refers to a person's capacity to move around and engage in activities without significant limitations. Validated scales like the Oswestry Disability Index are tools used to assess a person's level of disability and how it affects their ability to perform daily activities. These scales often consist of a series of questions or tasks that help quantify the extent of functional impairment, and the results can provide valuable insights into the individual's mobility limitations [23].

Assessment of activities of daily living (ADLs) and instrumental activities of daily living (IADLs): Activities of daily living are basic self-care tasks necessary for daily life, such as eating, bathing, dressing, and using the restroom. Instrumental activities of daily living are more complex activities essential for living independently, such as cooking, shopping, managing finances, and using transportation. Assessing a person's ability to perform these tasks helps gauge their overall functional independence and highlights areas where improvements are needed [24].

Monitoring patients' ability to perform tasks such as walking, bending, and lifting: The ability to walk, bend, lift objects, and perform other physical tasks is crucial for maintaining an active lifestyle and participating in various activities. Monitoring these capabilities provides insight into a person's progress and helps healthcare professionals tailor rehabilitation or treatment plans to address specific challenges and improve overall physical functioning [25].

Analysis of the impact of vertebral height restoration on physical function and overall independence: Vertebral height restoration refers to procedures or interventions to correct vertebral deformities, often associated with conditions like osteoporosis or spinal fractures. Analysing how such interventions impact physical function and independence involves assessing whether restoring vertebral height leads to improved mobility, reduced pain, and an enhanced ability to carry out daily tasks. This analysis can involve a combination of clinical evaluations, patient-reported outcomes, and objective measurements [26].

Radiological evaluation of vertebral height restoration

Importance of Radiological Assessment in Evaluating Treatment Success

Radiological evaluation involves the use of medical imaging techniques, such as X-rays, CT scans, or MRIs, to visualise and analyse the internal structures of the body. In the context of balloon kyphoplasty, radiological assessment is crucial for several reasons [27].

Objective measurement: Radiological imaging provides quantitative measurements of the restored vertebral height. This is important because it allows healthcare professionals to objectively assess how the procedure has successfully corrected the compression fracture. Objective measurements are essential for tracking progress and determining the effectiveness of the treatment [28].

Visual evidence: Radiological images offer visual evidence of the outcomes of the balloon kyphoplasty procedure. This evidence is invaluable for both healthcare providers and patients. It allows them to see the changes in the fractured vertebra and how well the restoration has been achieved. Visual evidence enhances understanding and communication between the medical team and the patient [29].

Treatment planning: Radiological assessment aids in treatment planning by providing detailed information about the location and extent of the compression fracture. This information guides healthcare professionals in determining the appropriate size of the balloon and the optimal placement of the bone cement. Accurate planning contributes to the overall success of the procedure [30].

Follow-up assessments: Radiological imaging is used for follow-up assessments after the procedure. These assessments help monitor the long-term stability of the restored vertebral height and detect any potential complications or issues. Regular follow-up imaging allows healthcare providers to intervene if there are signs of treatment failure or recurrence of the compression fracture [31].

Research and documentation: Radiological data gathered from various cases of balloon kyphoplasty contribute to medical research and documentation. These data can be analysed to identify trends, outcomes, and potential improvements in the procedure. It is a valuable resource for advancing medical knowledge and refining treatment techniques [32].

Research endeavours: The statement also emphasises that radiological assessment supports research endeavours related to balloon kyphoplasty. By collecting and analysing radiological data from various cases, researchers can better understand the factors contributing to successful outcomes and those that may lead to complications. This knowledge can drive advancements in the field and improve patient care [33].

Imaging Modalities for Assessing Vertebral Height Restoration

Various imaging modalities play a crucial role in assessing vertebral height restoration, allowing clinicians to monitor the progress of treatments and interventions to address vertebral fractures. These modalities provide valuable information about vertebral bodies' alignment, height, and structural integrity. The following imaging techniques are commonly employed for this purpose:

X-ray imaging, also known as radiography, is a commonly used and readily available imaging modality. It involves using ionising radiation to create images of the body's internal structures. In assessing vertebral height restoration, X-rays provide a quick and cost-effective way to visualise the overall alignment of the vertebral column and identify changes in vertebral height. By comparing X-ray images taken before and after treatment, clinicians can gauge the effectiveness of interventions in restoring vertebral height and alignment. However, X-rays may have limitations in assessing soft tissue changes and may provide less detailed information than other modalities [34].

Computed tomography scans: A CT scan utilises a series of X-ray images taken from different angles to create detailed cross-sectional images of the body. They offer three-dimensional visualisation, enabling accurate vertebral height and alignment measurements. They provide high-resolution images, allowing clinicians to precisely assess the degree of vertebral height restoration, detect residual deformities, and understand the spatial relationship between vertebrae. A CT scan is particularly valuable when requiring a more comprehensive assessment of vertebral fractures and height restoration [35].

Magnetic Resonance Imaging: An MRI is a non-ionising imaging technique that uses strong magnetic fields and radio waves to generate detailed images of the body's internal structures. While not typically used to measure vertebral height directly, MRI is valuable for evaluating soft tissue changes associated with vertebral fractures. It can provide information about the condition of the intervertebral discs, ligaments, and other supporting structures around the vertebral bodies. Additionally, MRI can assess the structural integrity of the vertebral body by detecting oedema, bone marrow changes, or signs of instability. This information is essential for understanding the overall health of the vertebral column and making informed decisions about treatment approaches [36].

Quantitative Measurement Techniques for Vertebral Height Restoration

Cobb angle measurement: The Cobb angle is widely used in spinal assessment to quantify the degree of curvature or deformity of the spine, such as scoliosis. In evaluating vertebral height restoration, the Cobb angle can be employed to assess changes in vertebral alignment post-treatment. This involves measuring the angle formed between two lines: one drawn along the vertebra’s superior (upper) endplate immediately above the treated level and the other drawn along the inferior (lower) endplate immediately below the treated level. By comparing Cobb angles before and after a procedure like balloon kyphoplasty, clinicians can objectively quantify any improvements in vertebral alignment and curvature [37].

Anterior and middle vertebral height measurements: Radiographic images allow measuring both the anterior (front) and middle vertebral heights. These measurements offer valuable insights into the extent of vertebral height restoration and its impact on overall spinal alignment. By assessing the anterior and middle heights of the treated vertebra, clinicians can determine how effectively the procedure has restored the vertebral body's height and, consequently, its structural integrity [28].

Assessment of vertebral alignment: Radiographs and advanced imaging techniques, such as CT scans and MRI, comprehensively evaluate vertebral alignment. Specific measurements like the sagittal index and wedge angle play a key role in assessing the success of vertebral height restoration procedures. The sagittal index measures the spine’s alignment in the sagittal plane (from the side view), helping to quantify the correction achieved in the kyphotic (forward curvature) deformity. The wedge angle measures the degree of deformity in vertebral bodies, giving clinicians an indication of how well the vertebral height restoration has corrected the deformity [38].

Factors influencing vertebral height restoration

Patient-Related Factors Affecting Outcome

Age: Age is a critical factor in bone health and healing capacity. Younger patients tend to have a more active bone metabolism, which can contribute to better outcomes in vertebral height restoration procedures. Their bones have a higher regenerative potential, making them more responsive to treatments to restore vertebral height [39].

Bone density: Bone density refers to the amount of mineral content in bones, and it's a key indicator of bone strength. Low bone density, often seen in conditions like osteoporosis, can compromise the structural integrity of the vertebral body. This weakened bone structure may affect the success of vertebral height restoration and the restored vertebra's long-term stability [40].

Comorbidities: Underlying health conditions can impact bone health and healing. Conditions such as diabetes, smoking, and cardiovascular diseases can impair blood circulation, decrease the supply of nutrients to bones, and interfere with the body's natural healing processes. These factors might negatively influence the outcome of vertebral height restoration procedures [41].

Fracture Type and Severity

Fracture type: The type of vertebral fracture plays a significant role in determining the success of height restoration. Different fracture types, such as wedge fractures (where the front of the vertebral body collapses) or crush fractures (where the vertebral body collapses uniformly), require tailored approaches for restoration. The choice of treatment and techniques can vary based on the specific fracture type [1].

Fracture severity: The severity of the vertebral fracture is another crucial factor. The degree of vertebral collapse and the presence of vertebral body fragments can impact the ability to restore vertebral height. Severe collapses or fractures with fragmented bone might present challenges in achieving optimal height restoration, as there might be limitations to the extent of restoration that can be achieved safely [1].

Procedural Factors Influencing Vertebral Height Restoration

Balloon inflation pressure and volume: During balloon kyphoplasty, a balloon is inserted into the fractured vertebral body and inflated to create a void or cavity. This void is then filled with bone cement to restore vertebral height. The pressure and volume of balloon inflation play a crucial role in this process [42].

Impact on vertebral height restoration: Higher inflation pressure can improve vertebral height restoration. When the balloon is inflated with higher pressure, it can more effectively create space within the compressed vertebral body, helping to restore the original vertebral height [1].

Balancing with complications: However, higher pressure must be balanced with the risk of complications. Excessive pressure could cause fractures of the surrounding cortical bone or even lead to cement leakage into undesirable areas. Careful consideration is necessary to achieve the desired height restoration without causing harm [43].

Integrity of surrounding cortical bone: Controlled inflation is essential to ensuring the surrounding cortical bone remains intact. This is crucial for the stability of the vertebral body. The goal is to restore height while avoiding any damage to the bone that might compromise its structural integrity [44].

Cement distribution and augmentation: The void is filled with bone cement after inflating and deflating the balloon. Proper distribution and augmentation of the cement are essential for achieving lasting height restoration and providing stability to the treated vertebra [45].

Sustainable height restoration: The cement must be distributed evenly within the vertebral body to maintain the restored height over time. Uneven distribution could lead to focal areas of load-bearing, potentially causing the vertebra to collapse again [46].

Structural support and stability: Adequate cement augmentation refers to using an appropriate amount of cement to provide structural support to the vertebral body. This augmentation helps prevent further collapse and provides stability to the fractured vertebra. It reinforces the weakened bone [47].

Long-term effects and sustainability of vertebral height restoration

Evidence on Long-Term Maintenance of Vertebral Height

Long-term studies: The paragraph highlights the significance of conducting long-term studies to assess the effectiveness of balloon kyphoplasty. Short-term studies might provide initial insights into the immediate benefits of the procedure. Still, long-term studies are essential to understand how well the treatment holds up over extended periods, typically several years [48].

Sustainability of vertebral height restoration: The main goal of balloon kyphoplasty is to restore the vertebral height lost due to compression fractures. This height restoration can alleviate pain, improve spinal alignment, and enhance overall quality of life. However, it's crucial to determine whether the restored vertebral height is maintained over the long term or if there is any gradual loss of height over time [49].

Insights into durability: By tracking patients who have undergone balloon kyphoplasty over extended periods, researchers can gain insights into the durability of the procedure's outcomes. This refers to how well the effects of the procedure last over time. Long-term studies can reveal whether the restored vertebral height remains stable and whether there is a reduced risk of subsequent vertebral collapses, which is crucial for ensuring the long-term effectiveness of the treatment [5].

Vertebral height maintenance and prevention of subsequent collapses: The two specific aspects that long-term studies focus on are, first, how well the vertebral height restoration achieved through balloon kyphoplasty is maintained over the years. Secondly, these studies collapse in the treated area [50-51].

Impact of Vertebral Height Restoration on Adjacent Level Fractures

The impact of vertebral height restoration on adjacent-level fractures is an essential consideration in spinal health and orthopaedics. This phenomenon is particularly relevant in treating vertebral compression fractures, often resulting from osteoporosis. Vertebral compression fractures involve the collapse of vertebral bodies, leading to a reduction in vertebral height. Restoring vertebral height is a primary goal of treatment. It is typically achieved through procedures like vertebroplasty or kyphoplasty, where the fractured vertebra is stabilised and sometimes filled with bone cement to regain its height [50].

Biomechanical changes: The spine functions as a biomechanical unit, distributing forces evenly along its various segments. When one vertebral body is compressed or fractured, the load it usually carries is transferred to the neighbouring vertebrae. When a fractured vertebra is restored to its original height, it can alter the distribution of loads on the adjacent levels [52].

Increased stress on adjacent vertebrae: Restoring vertebral height alters the spine’s natural curvature, affecting the distribution of forces across the spine's segments. This can increase stress and load on the vertebrae adjacent to the treated level. The sudden increase in load on these adjacent vertebrae may make them more susceptible to fractures, particularly if they are weakened due to conditions like osteoporosis [53].

Risk of fractures: The increased load on adjacent vertebrae due to vertebral height restoration can contribute to a higher risk of fractures in these regions. The altered biomechanics, coupled with the potential weakening of the bone due to osteoporosis or other factors, can create an environment where fractures are more likely to occur [54].

Long-term assessment: The potential impact of height restoration on adjacent vertebral levels underscores the importance of long-term assessment and follow-up. Patients undergoing vertebral height restoration procedures should be closely monitored to determine if any fractures or structural changes develop in the adjacent vertebrae. This ongoing assessment is crucial for understanding the procedure's impact on the spine's overall health [49].

Patient management strategies: The information from the long-term assessment can guide patient management strategies. If a pattern of fractures in adjacent vertebrae becomes evident, healthcare providers may need to consider alternative treatment approaches or provide additional interventions to strengthen the adjacent vertebral levels [1].

Consideration of Re-fracture Rates and Follow-Up Assessments

Treating vertebral compression fractures, particularly in osteoporotic patients, involves various techniques, such as balloon kyphoplasty. This procedure aims to alleviate pain, restore vertebral height, and enhance patients’ overall quality of life. However, re-fractures post-balloon kyphoplasty have emerged as a notable concern within the medical community. Re-fractures refer to new vertebral compression fractures in segments adjacent to or near the previously treated vertebrae [55].

While effective in the short term, balloon kyphoplasty must be evaluated regarding its long-term outcomes. This is where long-term follow-up assessments come into play. These assessments involve monitoring patients over an extended period, usually several years, to track their progress and identify any potential complications or issues that may arise beyond the immediate postoperative period [48].

Identifying underlying causes: Long-term follow-up evaluations provide clinicians with valuable data to better understand the underlying causes of re-fractures. Factors such as changes in bone density, altered biomechanics, and potential limitations of the kyphoplasty procedure itself can contribute to re-fracture risk. By observing patterns in re-fracture occurrences, medical professionals can gain insights into the specific factors contributing to this phenomenon [56].

Refining treatment protocols: The insights gained from long-term follow-up assessments can inform the refinement of treatment protocols. If specific patient demographics or procedural techniques are associated with higher refracture rates, adjustments can be made to the treatment approach. This might involve tailoring the procedure to address specific patient characteristics or using additional interventions to reduce re-fracture risk [57].

Minimising recurrence: The ultimate goal of tracking refracture rates through long-term follow-up is to minimise recurrence. By identifying high-risk patients or scenarios, medical practitioners can implement preventive measures or modify post-operative care to mitigate the chances of re-fractures. This contributes to improving patient outcomes and overall treatment success [58].

Clinical decision-making: Long-term follow-up assessments provide critical data for clinical decision-making. When recommending balloon kyphoplasty or other interventions for vertebral compression fractures, healthcare providers can use the accumulated knowledge of re-fracture rates to guide their recommendations. This ensures that patients receive treatments that are both effective and tailored to their individual needs [59].

Patient counselling: Informed patient consent and counselling are essential to medical practice. When patients are considering balloon kyphoplasty, discussing the potential for re-fractures and presenting data from long-term follow-up studies empowers them to make well-informed decisions about their treatment. Realistic expectations can be set, and patients can actively participate in their healthcare journey [60].

Treatment strategy refinement: Medicine constantly evolves, and treatment strategies must adapt to emerging evidence and insights. Long-term follow-up assessments contribute to the ongoing refinement of treatment strategies for vertebral compression fractures. As more data accumulate, medical professionals can fine-tune procedures, postoperative care plans, and patient selection criteria to optimise outcomes [61].

Functional recovery and quality of life

Relationship between Vertebral Height Restoration and Functional Improvement

Vertebral height restoration: Balloon kyphoplasty is a minimally invasive surgical procedure used to treat vertebral compression fractures, often due to osteoporosis or trauma. During this procedure, a small balloon is inserted into the fractured vertebral body and inflated to create a cavity. This cavity is then filled with a cement-like material, which helps stabilise the vertebral body and restore its height. The restoration of vertebral height is critical to this procedure [49].

Functional improvement: Functional improvement refers to enhancing a patient's ability to perform everyday activities, maintain mobility, and experience a higher quality of life. Vertebral compression fractures can lead to various issues, including severe pain, limited mobility, and a decreased ability to perform routine tasks. This is often due to the loss of vertebral height and the resulting spine deformity, which can negatively affect biomechanics and overall spinal alignment [61].

Relationship between vertebral height restoration and functional improvement: The relationship between vertebral height restoration and functional improvement is multifaceted and can be understood through the following points:

Pain reduction: Vertebral compression fractures can cause significant pain due to nerve impingement, inflammation, and instability. B-balloon kyphoplasty can alleviate pain by restoring vertebral height and stabilising the fracture, allowing patients to move more comfortably and engage in daily activities without constant discomfort [62].

Spinal alignment: Vertebral compression fractures often result in kyphosis, a forward curvature of the spine that can lead to a hunched or stooped posture. Balloon kyphoplasty can improve spinal alignment by restoring vertebral height and reducing kyphotic deformities. This improved alignment can lead to better weight distribution along the spine, reducing the risk of further fractures and improving overall biomechanics [60].

Enhanced mobility: Restoring spinal alignment and reducing deformities can directly impact a patient's mobility. A more aligned spine allows for more efficient movement as it restores the natural balance and coordination of the musculoskeletal system. Patients who undergo successful balloon kyphoplasty often experience enhanced mobility, allowing them to perform activities that were once difficult or impossible due to pain and limited movement [63].

Quality of life: Functional improvement from vertebral height restoration can profoundly impact a patient's quality of life. Moving more freely, engaging in social activities, and performing daily tasks without hindrance contribute to improved overall well-being and mental outlook [49].

Assessment Tools for Evaluating Functional Recovery

Oswestry Disability Index (ODI): The Oswestry Disability Index is a questionnaire used to assess the impact of back pain on an individual's daily life and functional abilities. It consists of ten sections, each focusing on a different aspect of everyday activities, such as pain intensity, lifting, walking, sitting, standing, sleeping, social life, travelling, and self-care. Patients rate their level of disability or limitation in each section, and the scores are then totalled to provide an overall disability score. The score is usually expressed as a percentage, with higher percentages indicating a more significant disability. The ODI helps gauge how much back pain affects a person's ability to perform various tasks [64].

Roland-Morris Disability Questionnaire: Similar to the ODI, the Roland-Morris Disability Questionnaire is another self-reported questionnaire that assesses functional limitations due to back pain. It consists of 24 statements related to daily activities, and patients are asked to check off the statements that apply to them. The number of checked items is then used to calculate a disability score, with higher scores indicating more severe functional limitations. This tool focuses on how back pain impacts a person's ability to carry out daily activities [23].

Physical performance measures: Physical performance measures objectively assess a patient's physical capabilities and mobility. These tests provide valuable information about a patient's functional recovery and progress. Two commonly used physical performance tests are the timed up and go (TUG) test, which evaluates a person's mobility and balance. The individual is timed as they stand up from a chair, walk a short distance, turn around, walk back to the chair, and sit down again. The time taken for the entire task provides insights into their mobility and balance [65], as does the six-minute walk test, which measures a person's endurance and cardiovascular fitness. Patients are instructed to walk as far as they can in six minutes. The distance covered indicates their functional capacity and endurance [66]. These physical performance measures help assess the functional limitations caused by back pain and the progress made during rehabilitation and recovery. They provide objective data that can be tracked over time to monitor improvements.

Impact on activities of daily living and overall quality of life

Vertebral Height Restoration and Activities of Daily Living

Activities of daily living (ADLs) refer to the routine tasks that individuals typically perform as part of their daily self-care and functional independence. These activities include bathing, dressing, eating, grooming, toileting, and mobility-related actions such as walking, standing, and sitting. Vertebral height restoration can have a significant impact on ADLs in the following ways:

Reduced pain: Vertebral fractures, often caused by conditions like osteoporosis, can result in severe pain and discomfort, particularly during movement. By restoring vertebral height through medical interventions like vertebroplasty or kyphoplasty, the compression on nerves and surrounding tissues is alleviated, reducing pain. This reduction in pain enables individuals to perform ADLs with greater ease and comfort [1].

Enhanced mobility: Vertebral fractures can compromise spinal alignment and overall mobility. When vertebral height is restored, spinal alignment improves overall posture and mobility. This improvement in mobility is crucial for performing activities such as walking, bending, and reaching, which are essential for independent living [67].

Improved comfort: Activities like dressing, grooming, and bathing can become challenging or painful due to vertebral fractures. Restoring vertebral height helps individuals perform these activities more comfortably and with reduced limitations [1].

Vertebral Height Restoration and Quality of Life

Psychological well-being: Chronic pain and physical limitations resulting from vertebral fractures can significantly negatively impact an individual's psychological well-being. Vertebral height restoration improves mental health outcomes by reducing pain and improving mobility. Reduced pain is often associated with decreased anxiety, depression, and psychological distress [68].

Social interactions: Pain and physical limitations can lead to social isolation and a reduced ability to engage in social activities. As vertebral height is restored and mobility improves, individuals are more likely to participate in social interactions, hobbies, and recreational activities, enhancing social connectedness and overall life satisfaction [69].

Independence: The ability to independently perform ADLs is closely linked to an individual's independence and autonomy. Vertebral height restoration directly supports independence by allowing individuals to engage in tasks without relying heavily on assistance from others [70].

Functional capacity: Individuals can engage in a broader range of physical activities with improved spinal alignment and reduced pain. This can lead to a sense of accomplishment and improved self-esteem as they regain the ability to participate in activities they may have previously avoided due to pain or limitations [71].

Complications and adverse events

Cement leakage (extravasation): One potential complication of balloon kyphoplasty is the leakage of the cement-like material into surrounding tissues. The cement is meant to stay within the vertebral body to provide stability, but there's a risk that it could leak into nearby structures such as the spinal canal or blood vessels. Cement leakage into the spinal canal can lead to spinal cord compression and neurological deficits, while leakage into blood vessels might cause embolisms or other vascular issues [72].

Fracture extension: During balloon kyphoplasty, a balloon is inflated within the collapsed vertebra to create a void before injecting the cement. If the balloon is overinflated or there's excessive pressure applied, it can potentially cause the fracture to extend further, leading to additional vertebral damage [72].

Infection: As with any procedure involving inserting instruments into the body, there's a risk of infection. In the case of balloon kyphoplasty, there's a potential for infection at the puncture site where the instruments are inserted, as well as within the treated vertebral body. Infections can lead to pain, inflammation, and other complications [73].

Neurological complications: The injection of cement during the procedure can potentially migrate to unintended areas. If cement migrates and compresses nearby nerves or the spinal cord, it can result in nerve injury or irritation. This may lead to neurological symptoms such as pain, numbness, weakness, or paralysis [74].

Vascular events: Another risk associated with balloon kyphoplasty is the potential for vascular events, such as embolisms. If cement leaks into blood vessels, it can form clots that may travel to other parts of the body, causing blockages and potentially leading to severe complications like stroke or pulmonary embolism [75].

Risk Factors for Adverse Events

Bone quality: Poor bone density or quality increases the risk of cement leakage. Bone quality plays a crucial role in the success of orthopaedic procedures. When performing a procedure like cement augmentation (for example, vertebroplasty or kyphoplasty), where cement is injected into bones to provide support or stability, poor bone density or quality can lead to several complications. If the bone is weak or porous, there is a higher chance of the cement not adhering correctly to the bone or leaking into adjacent tissues. This leakage can result in pain, inflammation, and nerve compression. Preoperative assessment of bone quality through imaging techniques like DEXA scans is essential to determine the procedure’s suitability for the patient [76].

Patient factors: Comorbidities like diabetes, vascular disease, or a compromised immune system can elevate risks. Patients with underlying health conditions are at an increased risk of experiencing complications during orthopaedic procedures. Diabetes, vascular diseases, and compromised immune systems can negatively impact the body's ability to heal and respond to the procedure. For instance, diabetes can affect blood circulation and impair wound healing, making it more likely for infections to develop post-surgery. Vascular diseases can lead to poor blood flow, which may hinder the delivery of necessary nutrients to the surgical site. A compromised immune system can weaken the body's defence mechanisms against infections. Thus, carefully considering a patient's medical history and comorbidities is crucial in assessing the overall risk and deciding whether the procedure is appropriate [77].

Procedural factors: Inadequate visualisation during the procedure can contribute to complications. Procedural factors directly related to how the surgery is performed influence the risk of adverse events. Inadequate visualisation refers to the surgeon's ability to see and assess the surgical site. Accurate cement placement is critical to avoid leakage and ensure proper stability during cement augmentation. If the surgeon needs help visualising the target area due to anatomical obstacles or poor imaging techniques, there's a higher likelihood of errors. Inaccurate placement can lead to complications such as cement leakage, nerve damage, or improper stabilisation of the bone. Therefore, advanced imaging techniques like fluoroscopy or intraoperative CT scans and surgeon experience are vital in minimising procedural risks [78].

Strategies for Minimizing Complications and Enhancing Safety

Image guidance: Precise imaging, typically achieved through techniques like fluoroscopy (real-time X-ray) or CT (computed tomography), is crucial for ensuring the accurate placement of instruments and cement. This step allows the surgeon to visualise the targeted vertebra and surrounding structures, guiding the needle and cement placement with high accuracy. Visualising the procedure in real-time can minimise the risk of inadvertently damaging adjacent structures [79].

Balloon inflation control: In procedures like kyphoplasty, where a balloon is used to create a cavity before cement injection, careful monitoring of balloon inflation is essential. Overinflation of the balloon can lead to excessive vertebral body compression, increasing the risk of fractures or other complications. Proper monitoring and controlled inflation ensure the cavity is created safely and appropriately [12].

Cement augmentation technique: The distribution and injection technique of the cement plays a significant role in the success and safety of the procedure. Ensuring a uniform and controlled distribution of cement within the vertebral body reduces the risk of cement leakage into surrounding tissues. The choice of cement viscosity, injection pressure, and specific tools to optimise distribution can all minimise the risk of complications [80].

Patient selection: Rigorous patient evaluation is crucial for identifying individuals who are suitable candidates for these procedures and those who may be at a higher risk of complications. Factors such as bone quality, extent of vertebral compression, overall health, and potential contraindications are carefully considered. Patients with severe osteoporosis, multiple vertebral fractures, or certain medical conditions may require extra caution or alternative treatment options [81].

Surgeon experience: The skill and experience of the operating surgeon significantly influence the safety and success of vertebral cement augmentation procedures. Experienced surgeons are more adept at accurately guiding instruments, interpreting imaging, and making real-time adjustments to the procedure as needed. Their expertise reduces the likelihood of procedural errors, improves patient outcomes, and minimises the risk of complications [82].

Future directions and research implications

Emerging Technologies and Techniques in Vertebral Height Restoration

Biodegradable implants: Biodegradable implants represent a significant leap forward in orthopaedic medicine. These implants are made from materials that naturally degrade over time while promoting bone healing and regeneration. For vertebral height restoration, biodegradable implants can be designed to provide initial structural support and stability while gradually being absorbed by the body as new bone tissue forms. This eliminates the need for additional surgery to remove the implant once healing is complete. The biodegradable nature of these implants reduces the risk of long-term complications and can improve patient outcomes [83].

3D printing: 3D printing technology has revolutionised the field of medicine by allowing for the creation of patient-specific implants and devices. In vertebral height restoration, 3D printing enables the production of customised implants that precisely fit the patient's anatomy. These implants can be designed to match the exact dimensions of the fractured vertebra, ensuring optimal restoration of vertebral height. The ability to create personalised implants reduces the risk of implant misfit, enhances stability, and promotes better overall patient outcomes [84].

Minimally invasive navigation: Minimally invasive techniques are designed to reduce tissue trauma, pain, and patient recovery time. Advanced navigation technologies play a crucial role in these procedures. Imaging modalities like fluoroscopy, CT scans, and MRI can be combined with navigation systems for real-time surgical site visualization. Surgeons can use this information to guide instruments and cement placement accurately. This precision minimises the risk of damaging healthy tissue and ensures optimal distribution of bone cement within the fractured vertebra, enhancing stability and promoting proper healing [85].

Areas Requiring Further Research and Investigation

Long-term outcomes: One area requiring further research is the long-term outcomes of vertebral augmentation procedures, such as balloon kyphoplasty or vertebroplasty. While these procedures have shown promising results in terms of immediate pain relief and vertebral height restoration, there is a need for more comprehensive studies that track patients over extended periods. These studies aim to understand the durability of vertebral height restoration and assess how patients' conditions evolve years after the initial procedure. It is important to determine whether the benefits of these procedures are sustained over the long term and to identify any potential complications or issues that might arise years after the initial treatment.

Comparative studies: Another critical avenue of research is conducting comparative assessments of different vertebral augmentation techniques. Various methods and materials are used in vertebral augmentation, each with advantages and limitations. Comparative studies would directly compare the efficacy, safety, and long-term benefits of different techniques, such as balloon kyphoplasty and vertebroplasty, assessing factors such as pain relief, vertebral height restoration, and post-procedure complications. Such studies help clinicians decide which technique might be best suited for specific patient populations and conditions.

Adjunctive therapies: Exploring the potential of combined therapies is another area that requires attention. One avenue is investigating the use of bone growth factors or other regenerative agents alongside vertebral augmentation procedures. These additional treatments could enhance bone healing and improve the overall outcomes of the procedures. This area of research involves studying the interactions between different treatments, understanding their synergistic effects, and evaluating their impact on patient recovery and long-term outcomes.

Fracture prevention strategies: Osteoporosis is a common underlying condition in patients who undergo vertebral augmentation procedures, as vertebral fractures often occur due to weakened bones. Research into fracture prevention strategies is crucial to minimising the risk of subsequent fractures in these patients. This could involve studying medications, lifestyle modifications, exercise regimens, and nutritional interventions that can enhance bone density and strength. Understanding how to effectively prevent further fractures after the initial vertebral augmentation procedure is essential for maintaining patients' overall spinal health.

Potential Improvements in Patient Selection and Procedural Optimization

Advanced imaging: Utilising advanced imaging techniques, such as MRI, in assessing bone quality can provide valuable insights into the structural integrity of the vertebrae. An MRI can offer detailed information about bone density, microarchitecture, and the presence of any underlying pathology that might affect the success of vertebral height restoration procedures. By incorporating these advanced imaging modalities, clinicians can make more informed decisions about which patients are suitable candidates for the procedure and predict the potential outcomes more accurately.

Predictive models: Developing predictive models involves leveraging data from various sources, including patient demographics, bone quality assessments, and fracture characteristics. By analysing these factors collectively, it becomes possible to create models to predict the likely success of vertebral height restoration for a given patient. These models could aid physicians in making evidence-based decisions on patient selection and treatment approaches. By identifying patients most likely to benefit from the procedure, healthcare resources can be allocated more efficiently.

Individualised treatment: Every patient is unique, and factors like age, bone density, overall health, and the nature of the vertebral fracture can vary significantly. Tailoring treatment plans based on patient-specific factors allows for a more personalised approach to vertebral height restoration. This could involve choosing the most appropriate technique, implant, or material for each patient and adjusting parameters such as implant size and placement to optimise the outcomes. The chances of successful vertebral height restoration can be increased by customising the procedure for individual patients.

Minimising complications: Continuous refinement of procedural techniques and instruments is crucial for reducing the risk of complications associated with vertebral height restoration procedures. As more experience is gained and new insights are acquired, clinicians can identify potential sources of complications and develop strategies to mitigate them. This might involve improving surgical instruments for better precision, optimising the procedure steps to minimise tissue damage, and enhancing post-operative care protocols to promote faster recovery and reduce the risk of infection or other adverse events.

## Conclusions

In conclusion, this comprehensive review has shed light on the intricate realm of vertebral height restoration after balloon kyphoplasty in vertebral compression fractures. A profound understanding has emerged by exploring procedural intricacies, radiological assessments, clinical outcomes, and influencing factors. Beyond pain relief, restoring vertebral height is critical to improved spinal alignment, enhanced functional recovery, and a better quality of life. This review underscores the transformative impact of balloon kyphoplasty, not just as a medical intervention but as a gateway to renewed independence and well-being for those grappling with vertebral compression fractures. As medical science advances and research endeavours continue, the significance of vertebral height restoration remains at the forefront of innovative approaches, ultimately offering a brighter horizon for patients seeking relief, mobility, and the restoration of a life less burdened by pain.

## References

[1] Alexandru D, So W (2012). Evaluation and management of vertebral compression fractures. Perm J.

[2] Donnally III CJ, DiPompeo CM, Varacallo M (2023). Vertebral Compression Fractures. https://www.ncbi.nlm.nih.gov/books/NBK448171/.

[3] Taylor RS, Fritzell P, Taylor RJ (2007). Balloon kyphoplasty in the management of vertebral compression fractures: an updated systematic review and meta-analysis. Eur Spine J.

[4] Medical Advisory Secretariat (2004). Balloon kyphoplasty: an evidence-based analysis. Ont Health Technol Assess Ser.

[5] Marcucci G, Brandi ML (2010). Kyphoplasty and vertebroplasty in the management of osteoporosis with subsequent vertebral compression fractures. Clin Cases Miner Bone Metab.

[6] Voggenreiter G (2005). Balloon kyphoplasty is effective in deformity correction of osteoporotic vertebral compression fractures. Spine (Phila Pa 1976).

[7] Teyssédou S, Saget M, Pries P (2014). Kyphopasty and vertebroplasty. Orthop Traumatol Surg Res.

[8] (2023). What is a kyphoplasty procedure?. https://www.greateraustinpain.com/blog/what-is-a-kyphoplasty-procedure.

[9] Saliou G, Rutgers DR, Kocheida EM, Langman G, Meurin A, Deramond H, Lehmann P (2010). Balloon-related complications and technical failures in kyphoplasty for vertebral fractures. AJNR Am J Neuroradiol.

[10] Krüger A, Oberkircher L, Kratz M, Baroud G, Becker S, Ruchholtz S (2012). Cement interdigitation and bone-cement interface after augmenting fractured vertebrae: a cadaveric study. Int J Spine Surg.

[11] Hu L, Sun H, Wang H, Cai J, Tao Y, Feng X, Wang Y (2019). Cement injection and postoperative vertebral fractures during vertebroplasty. J Orthop Surg Res.

[12] Heini PF, Orler R (2004). Kyphoplasty for treatment of osteoporotic vertebral fractures. Eur Spine J.

[13] Marcucci G, Brandi ML (2009). Vertebroplasty and balloon kyphoplasty in osteoporosis: friends or foes?. Clin Cases Miner Bone Metab.

[14] Ma XL, Xing D, Ma JX, Xu WG, Wang J, Chen Y (2012). Balloon kyphoplasty versus percutaneous vertebroplasty in treating osteoporotic vertebral compression fracture: grading the evidence through a systematic review and meta-analysis. Eur Spine J.

[15] Masala S, Fiori R, Massari F, Simonetti G (2005). Kyphoplasty: indications, contraindications and technique. Radiol Med.

[16] Zambouri A (2007). Preoperative evaluation and preparation for anesthesia and surgery. Hippokratia.

[17] Nichol JR, Sundjaja JH, Nelson G (2023). Medical History. https://www.ncbi.nlm.nih.gov/books/NBK534249/.

[18] Whitney E, Alastra AJ (2023). Vertebral Fracture. https://www.ncbi.nlm.nih.gov/books/NBK547673/.

[19] Goldberg AL, Kershah SM (2010). Advances in imaging of vertebral and spinal cord injury. J Spinal Cord Med.

[20] Kim HS, Jeong ES, Yang MH, Yang SO (2018). Bone mineral density assessment for research purpose using dual energy X-ray absorptiometry. Osteoporos Sarcopenia.

[21] Firanescu CE, de Vries J, Lodder P (2018). Vertebroplasty versus sham procedure for painful acute osteoporotic vertebral compression fractures (VERTOS IV): randomised sham controlled clinical trial. BMJ.

[22] Borgström F, Olafsson G, Ström O, Tillman JB, Wardlaw D, Boonen S, Miltenburger C (2013). The impact of different health dimensions on overall quality of life related to kyphoplasty and non-surgical management. Osteoporos Int.

[23] Maughan EF, Lewis JS (2010). Outcome measures in chronic low back pain. Eur Spine J.

[24] Edemekong PF, Bomgaars DL, Sukumaran S, Schoo C (2023). Activities of Daily Living. https://www.ncbi.nlm.nih.gov/books/NBK470404/.

[25] Milanović Z, Pantelić S, Trajković N, Sporiš G, Kostić R, James N (2013). Age-related decrease in physical activity and functional fitness among elderly men and women. Clin Interv Aging.

[26] Yu D, Liu Z, Wang H, Yao R, Li F, Yang Y, Sun F (2021). Analysis on the effect of different surgical methods on the treatment of senile osteoporotic spinal compression fractures and the influencing factors of complications. Evid Based Complement Alternat Med.

[27] Zhang Z, Sejdić E (2019). Radiological images and machine learning: trends, perspectives, and prospects. Comput Biol Med.

[28] Hsu WE, Su KC, Chen KH, Pan CC, Lu WH, Lee CH (2019). The evaluation of different radiological measurement parameters of the degree of collapse of the vertebral body in vertebral compression fractures. Appl Bionics Biomech.

[29] Dalbayrak S, Onen MR, Yilmaz M, Naderi S (2010). Clinical and radiographic results of balloon kyphoplasty for treatment of vertebral body metastases and multiple myelomas. J Clin Neurosci.

[30] Wei P, Yao Q, Xu Y, Zhang H, Gu Y, Wang L (2019). Percutaneous kyphoplasty assisted with/without mixed reality technology in treatment of OVCF with IVC: a prospective study. J Orthop Surg Res.

[31] Panda A, Das CJ, Baruah U (2014). Imaging of vertebral fractures. Indian J Endocrinol Metab.

[32] Giordan E, Del Verme J, Pastorello G (2022). Treatment of thoracolumbar burst fractures: SpineJack vs. posterior arthrodesis-comparison of clinical and radiological outcomes. J Spine Surg.

[33] Guo J, Zhai W, Wei L (2021). Radiological and clinical outcomes of balloon kyphoplasty for osteoporotic vertebral compression fracture in patients with rheumatoid arthritis. J Orthop Surg Res.

[34] Health C for D and R (2023). Medical X-ray imaging. https://www.fda.gov/radiation-emitting-products/medical-imaging/medical-x-ray-imaging.

[35] (2023). How does a CT or CAT scan work?. https://www.medicalnewstoday.com/articles/153201.

[36] Berger A (2002). Magnetic resonance imaging. BMJ.

[37] Horng MH, Kuok CP, Fu MJ, Lin CJ, Sun YN (2019). Cobb angle measurement of spine from X-ray images using convolutional neural network. Comput Math Methods Med.

[38] Vialle R, Levassor N, Rillardon L, Templier A, Skalli W, Guigui P (2005). Radiographic analysis of the sagittal alignment and balance of the spine in asymptomatic subjects. J Bone Joint Surg Am.

[39] Ferrucci L, Baroni M, Ranchelli A, Lauretani F, Maggio M, Mecocci P, Ruggiero C (2014). Interaction between bone and muscle in older persons with mobility limitations. Curr Pharm Des.

[40] Sözen T, Özışık L, Başaran NÇ (2017). An overview and management of osteoporosis. Eur J Rheumatol.

[41] Jiao H, Xiao E, Graves DT (2015). Diabetes and its effect on bone and fracture healing. Curr Osteoporos Rep.

[42] Wardlaw D, Van Meirhaeghe J, Ranstam J, Bastian L, Boonen S (2012). Balloon kyphoplasty in patients with osteoporotic vertebral compression fractures. Expert Rev Med Devices.

[43] Zhang K, Shen Y, Ren Y, Zou D (2015). Prevention and treatment of bone cement-related complications in patients receiving percutaneous kyphoplasty. Int J Clin Exp Med.

[44] La Barbera L, Cianfoni A, Ferrari A, Distefano D, Bonaldi G, Villa T (2019). Stent-screw assisted internal fixation of osteoporotic vertebrae: a comparative finite element analysis on Saif technique. Front Bioeng Biotechnol.

[45] Yimin Y, Zhiwei R, Wei M, Jha R (2013). Current status of percutaneous vertebroplasty and percutaneous kyphoplasty--a review. Med Sci Monit.

[46] Krüger A, Baroud G, Noriega D, Figiel J, Dorschel C, Ruchholtz S, Oberkircher L (2013). Height restoration and maintenance after treating unstable osteoporotic vertebral compression fractures by cement augmentation is dependent on the cement volume used. Clin Biomech (Bristol, Avon).

[47] Kim HS, Kim SH, Ju CI, Kim SW, Lee SM, Shin H (2010). The role of bone cement augmentation in the treatment of chronic symptomatic osteoporotic compression fracture. J Korean Neurosurg Soc.

[48] Bouza C, Lopez T, Magro A, Navalpotro L, Amate JM (1995). Efficacy and safety of balloon kyphoplasty in the treatment of vertebral compression fractures: a systematic review. Database of Abstracts of Reviews of Effects (DARE): Quality-assessed Reviews.

[49] Mooney JH, Amburgy J, Self M, Agee BS, Schoel L, Pritchard PR, Chambers MR (2019). Vertebral height restoration following kyphoplasty. J Spine Surg.

[50] Wong CC, McGirt MJ (2013). Vertebral compression fractures: a review of current management and multimodal therapy. J Multidiscip Healthc.

[51] Tolba R, Bolash RB, Shroll J, Costandi S, Dalton JE, Sanghvi C, Mekhail N (2014). Kyphoplasty increases vertebral height, decreases both pain score and opiate requirements while improving functional status. Pain Pract.

[52] Luo J, Adams MA, Dolan P (2010). Vertebroplasty and kyphoplasty can restore normal spine mechanics following osteoporotic vertebral fracture. J Osteoporos.

[53] Tzermiadianos MN, Renner SM, Phillips FM (2008). Altered disc pressure profile after an osteoporotic vertebral fracture is a risk factor for adjacent vertebral body fracture. Eur Spine J.

[54] Kim MH, Lee AS, Min SH, Yoon SH (2011). Risk factors of new compression fractures in adjacent vertebrae after percutaneous vertebroplasty. Asian Spine J.

[55] Yang T, Liu S, Lv X, Wu X (2010). Balloon kyphoplasty for acute osteoporotic compression fractures. Interv Neuroradiol.

[56] Han S, Jang IT (2018). Analysis of adjacent fractures after two-level percutaneous vertebroplasty: is the intervening vertebral body prone to re-fracture?. Asian Spine J.

[57] Paskins Z, Bullock L, Crawford-Manning F (2021). Improving uptake of fracture prevention drug treatments: a protocol for development of a consultation intervention (iFraP-D). BMJ Open.

[58] Rodziewicz TL, Houseman B, Hipskind JE (2023). Medical Error Reduction and Prevention. https://www.ncbi.nlm.nih.gov/books/NBK499956/.

[59] Liu JT, Li CS, Chang CS, Liao WJ (2015). Long-term follow-up study of osteoporotic vertebral compression fracture treated using balloon kyphoplasty and vertebroplasty. J Neurosurg Spine.

[60] Van Meirhaeghe J, Bastian L, Boonen S, Ranstam J, Tillman JB, Wardlaw D (2013). A randomized trial of balloon kyphoplasty and nonsurgical management for treating acute vertebral compression fractures: vertebral body kyphosis correction and surgical parameters. Spine (Phila Pa 1976).

[61] Genev IK, Tobin MK, Zaidi SP, Khan SR, Amirouche FM, Mehta AI (2017). Spinal compression fracture management: a review of current treatment strategies and possible future avenues. Global Spine J.

[62] Imani F, Gharaei H, Rahimzadeh P, Saffarian Z (2012). Management of painful vertebral compression fracture with kyphoplasty in a sever cardio-respiratory compromised patient. Anesth Pain Med.

[63] Bettany-Saltikov J, Turnbull D, Ng SY, Webb R (2017). Management of spinal deformities and evidence of treatment effectiveness. Open Orthop J.

[64] Vianin M (2008). Psychometric properties and clinical usefulness of the Oswestry Disability Index. J Chiropr Med.

[65] Podsiadlo D, Richardson S (1991). The timed "up & go": a test of basic functional mobility for frail elderly persons. J Am Geriatr Soc.

[66] Matos Casano HA, Anjum F (2023). Six-Minute Walk Test. https://www.ncbi.nlm.nih.gov/books/NBK576420/.

[67] Joo Y, Lee PB, Nahm FS (2011). Spontaneous height restoration of vertebral compression fracture - a case report. Korean J Pain.

[68] Lasaite L, Krasauskiene A (2009). Psychological state, quality of life, and body composition in postmenopausal women with osteoporosis in Lithuania. Arch Osteoporos.

[69] National Academies of Sciences, Engineering Engineering, and Medicine (2020). Risk and protective factors for social isolation and loneliness. Social Isolation and Loneliness in Older Adults: Opportunities for the Health Care System.

[70] Pollock A, Baer G, Campbell P (2014). Physical rehabilitation approaches for the recovery of function and mobility following stroke. Cochrane Database Syst Rev.

[71] Vincent KR, Vincent HK (2012). Resistance exercise for knee osteoarthritis. PM R.

[72] Walter J, Haciyakupoglu E, Waschke A, Kalff R, Ewald C (2012). Cement leakage as a possible complication of balloon kyphoplasty--is there a difference between osteoporotic compression fractures (AO type A1) and incomplete burst fractures (AO type A3.1)?. Acta Neurochir (Wien).

[73] Shaibani A, Ali S, Bhatt H (2007). Vertebroplasty and kyphoplasty for the palliation of pain. Semin Intervent Radiol.

[74] Kim HJ, Park SH, Shin HY, Choi YS (2014). Brachial plexus injury as a complication after nerve block or vessel puncture. Korean J Pain.

[75] Rodrigues DM, Cunha Machado DP, Campainha Fernandes SA, Paixão Barroso AM (2019). Pulmonary cement embolism following balloon kyphoplasty: the impact of a procedural complication in a new era for lung cancer management. Mol Clin Oncol.

[76] Gao X, Du J, Gao L, Hao D, Hui H, He B, Yan L (2022). Risk factors for bone cement displacement after percutaneous vertebral augmentation for osteoporotic vertebral compression fractures. Front Surg.

[77] Chávez-Reyes J, Escárcega-González CE, Chavira-Suárez E (2021). Susceptibility for some infectious diseases in patients with diabetes: the key role of glycemia. Front Public Health.

[78] Chung KC, Kotsis SV (2012). Complications in surgery: root cause analysis and preventive measures. Plast Reconstr Surg.

[79] Campbell DH, McDonald D, Araghi K, Araghi T, Chutkan N, Araghi A (2021). The clinical impact of image guidance and robotics in spinal surgery: a review of safety, accuracy, efficiency, and complication reduction. Int J Spine Surg.

[80] Guarnieri G, Masala S, Muto M (2015). Update of vertebral cementoplasty in porotic patients. Interv Neuroradiol.

[81] LeBoff MS, Greenspan SL, Insogna KL, Lewiecki EM, Saag KG, Singer AJ, Siris ES (2022). The clinician's guide to prevention and treatment of osteoporosis. Osteoporos Int.

[82] Galivanche AR, Toombs C, Adrados M (2021). Cement augmentation of vertebral compression fractures may be safely considered in the very elderly. Neurospine.

[83] Amini AR, Wallace JS, Nukavarapu SP (2011). Short-term and long-term effects of orthopedic biodegradable implants. J Long Term Eff Med Implants.

[84] Bozkurt Y, Karayel E (2021). 3D printing technology; methods, biomedical applications, future opportunities and trends. J Mater Res Technol.

[85] Khoshnevisan A, Allahabadi NS (2012). Neuronavigation: principles, clinical applications and potential pitfalls. Iran J Psychiatry.

